# Contemporary Uses of Vilca (*Anadenanthera colubrina* var *cebil*): A Major Ritual Plant in the Andes

**DOI:** 10.3390/plants13172398

**Published:** 2024-08-27

**Authors:** Verónica S. Lema

**Affiliations:** 1 Institute of Anthropology of Cordoba, National Scientific and Technical Research Council of Argentina (CONICET), Yrigoyen 174, Cordoba 5000, Argentina; vslema@gmail.com; 2 School of Philosophy and Humanities, National University of Cordoba, Av. Haya de la Torre, Cordoba 5000, Argentina

**Keywords:** Andes, medicinal plants, psychoactives, therapeutics

## Abstract

*Vilca* or *cebil* (*Anadenanthera colubrina* var. *cebil*) is a species known for its psychoactive properties and its widespread use among the pre-Hispanic peoples who inhabited the southern Andean area (southern Peru, Bolivia, northern Chile and northwest Argentina). Studies on this species, as well as on medicinal, psychoactive, or magical plants in general, tend to consider its use in post-Spanish conquest times to be scarce or irrelevant in the Andes of South America. However, based on an in-depth review of the existing literature and on ethnobotanical research conducted in markets in Argentina, Bolivia, and Peru, this paper provides an updated overview affirming the continuity of the use of this species. The results indicate a significant diversity in terms of usage types, plant parts used, treatments, and conditions in which it is applied, along with new records of vernacular names. This paper also offers an interpretation from the perspective of Andean logics, highlighting the current therapeutic effectiveness of the seeds of this plant, facilitated through a series of “movements” that aim to restore the affected person’s health.

## 1. Introduction

### 1.1. Background

Globally, the American continent stands out for its vast array of plant species with psychoactive properties [1]. In South America specifically, a wide variety of plants and their preparations can alter human perception through their chemical components [2]. The knowledge about these plants in South America is also deeply rooted in history. The earliest evidence dates back approximately 4000 years, with archeological findings of *Anadenanthera colubrina* (Vell.) Brenan var. *cebil* (Griseb.) Altschul [synonym *Anadenanthera macrocarpa* (Benth.) Brenan; Fabaceae] seeds alongside pipes used for smoking them in northwest Argentina [3,4].

The genus *Anadenanthera* has an exclusively neotropical distribution from the West Indies to Argentina. *Anadenanthera* trees are feathery-foliaged; the leaves are bipinnately compound, with leaflets 9–8 mm. long. The trunks may be smooth or appended with mammillose projections. The flower heads are up to 20 mm in diameter and range from white or greenish white to orange–yellow. The pods are up to 35 cm long and up to 3 cm wide; they are more or less flat and unilocular, dehiscing along one suture only, brownish outside, containing 8–16 thin, flat orbicular and shiny brown or black seeds 10–20 mm in diameter. *Anadenanthera colubrina* primarily inhabits seasonally dry forests between 0 and 30 degrees south latitude. Known as *vilca* or *willka* in Peru, Bolivia, and Chile, and as *cebil* in Argentina, *A. colubrina* var. *cebil* has been extensively used over time. Similarly, its relative *A. peregrina*, known as *yopo*, *parica,* or *cohoba*, is found in Colombia, Venezuela, English Guyana, Brazil, and Paraguay. It was also introduced in pre-Hispanic times to the Greater Antilles [3,5,6].

The psychoactive effects of this genus of plants, regarded as visionary and entheogenic, are primarily due to the presence of dimethyltryptamine (DMT), 5-hydroxy-DMT or bufotenin, and 5-MeO-DMT, particularly in their seeds [2,3]. In the pre-Hispanic Andean world, *vilca* held significant cosmological, medicinal, and ritual importance. However, its use began to decline with the expansion of the Inca empire and was further suppressed by the Spanish conquistadors, leading to its near extinction, or very limited use, from the 18th century onward [7]. While this perspective is widely accepted, recent studies [8] suggest that the situation might be different than previously assumed.

**Figure 1:**
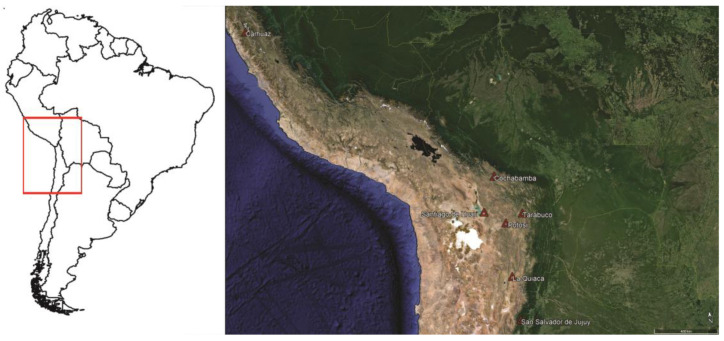
Map showing the Southern Andean Area and the countries it encompasses in South America (**left**), with traditional markets and fairs where *Anadenanthera* seeds were found during fieldwork (**right**).

This paper will present, on one hand, an exhaustive review of the existing literature for the Southern Andean Area—covering central and southern Peru, Bolivia, northern Chile, and northwest Argentina (Figure 1)—that documents the use of *vilca* or *cebil* from post-colonial times to the present (19th to 21st centuries). On the other hand, it will present the results of a survey conducted in various urban and peri-urban markets of northwest Argentina, Bolivia, and southern Peru to document the current uses of *Anadenanthera colubrina* var. *cebil*. Following the presentation of these results, the diversity of registered uses will be analyzed, culminating in a unified interpretation of the contemporary therapeutic applications of *vilca* or *cebil* seeds within the southern Andean cosmovision.

### 1.2. The Use of Anadenanthera colubrina var. cebil in Pre-Hispanic and Colonial Times (16th to 18th Centuries) in the Southern Andean Area

After its earliest presence in pipes 4000 years ago, remains of *vilca* or *cebil* seeds have continued to be detected in South America in various artifacts, including pipes, enemas, and items associated with snuffing, such as leather bags, tubes, and trays. These seeds have also been found as macro remains or as an ingredient in alcoholic beverages like *chicha de molle* (*Schinus molle* L.-Anacardiaceae) and *aloja*, a fermented drink made of different *Neltuma* [Fabaceae] species [3,4,9,10,11,12,13,14,15,16,17]. All these findings are confined to the Andean area, in particular northern Chile, northwest Argentina, central and southern Peru, and Bolivia. The use of *vilca* has a continuous record throughout the pre-Hispanic period, except during the expansion of the Inca or *Tawantinsuyo* empire. For this period, there is no direct archeological evidence of *vilca* seeds use; although, the consumption of this plant is mentioned in early chronicles of the region [7,18]

In pre-Hispanic archeological sites in northwest Argentina, remains of *cebil* seeds have been recovered, along with wood, either unmodified or used as raw material for the production of snuff trays [18,19,20,21]. Cebil wood has also been identified as fuel in cremation funerary contexts in that area [22]. According to early chronicles, its wood was used in the manufacture of furniture and artifacts [23]. Some authors, such as Gade [7], suggest that its wood may have been part of the ceremonial fire called *vilcanina*, used to offer sacrifices and oblations to the tutelary hills.

In the 17th century, several chroniclers of the central Andes—Cristóbal de Albornoz, Guaman Poma de Ayala, Bernabé Cobo, Polo de Ondegardo, and Ludovico Bertonio—documented the Inca customs of purging (oral and/or anal) with *vilca* seeds and its inclusion in *chicha* (fermented beverage), whether for medicinal, revitalizing, or visionary purposes [7,14,23].

Between the 16th and 18th centuries, the inhalation of *vilca* or *cebil* snuff was noted as a customary practice among the indigenous people of north central Argentina (provinces of Santiago del Estero and Córdoba), including its consumption during rituals to ask for rain [24]. During this period, there are also records of *cebil* seeds being collected as an indigenous tribute by a Spanish landowner in Santiago del Estero, as well as accounts of Spaniards and Creoles using these seeds in Córdoba during the early colonial period to forge alliances with indigenous chiefs [25,26,27]. In the 18th century, there are testimonies from Santiago del Estero of accusations of sorcery among indigenous women. One such account describes a remedy involving the ingestion of five ground *cebil* seeds in hot water to counteract an illness (*daño*) caused by one of them through *chicha* mixed with *chamico* (*Datura ferox* L.-Solanaceae) seeds [28].

These instances demonstrate the significant role of *vilca* or *cebil* in the pre-Hispanic and colonial history of the southern Andes. Therefore, the aim of this paper is, firstly, to examine whether this importance has been maintained since the 19th century by analyzing bibliographical background and conducting ethnobotanical fieldwork in traditional markets. Secondly, the aim of this paper is to offer a possible interpretation of the various uses of *Anadenanthera colubrina* var *cebil* seeds from the perspective of Andean therapeutic logics.

## 2. Materials and Methods

To survey the contemporary uses of *Anadenanthera colubrina* var. *cebil* in the Southern Andes (Figure 1), both an exhaustive literature review and ethnobotanical fieldwork in markets were conducted. The former focused on references to the use of *vilca* or *cebil* (including all recorded variants of both terms) [3,5] from the 19th century to the present. This step implied the use of various internet search engines and references to specialized books on plants used for medicinal, magical, and ritual purposes in the southern Andes. Ethnographic, anthropological, archeological, and ethnobotanical publications were explored, along with graduate and postgraduate theses from institutions in the countries within the study area. All recorded uses of the plant were documented, with a specific emphasis on those referring to the seeds. These uses were systematized according to the logics outlined by the original communities that utilize them, particularly focusing on traditional doctors and ritual specialists. Additionally, information from Wichí groups in the western sector of the Gran Chaco, close to the Andean area of northern Argentina, was included due to their widespread use of *cebil*. Other extra-Andean references were excluded, unless they documented a particular form of use not recorded in the primary area of interest for this paper.

The fieldwork was conducted between 2017 and 2019 in stalls selling medicinal products in Peru, Bolivia, and northwest Argentina (Figure 1). They are characterized by the sale of ingredients with traditional ritual and medicinal uses, including plants, animals, minerals, and various preparations made from these materials. The surveyed stalls are located in permanent markets and fairs held at different times of the year. The former include the Carhuaz Market (Ancash, Peru), Central Market, and Vicuñas Market in Potosi; Cochabamba Market and Tarabuco Market (Bolivia); and the old bus terminal market in San Salvador de Jujuy (Argentina). The fairs include the Manka Fiesta, usually held on the third Sunday of October in La Quiaca, Jujuy, Argentina, and the Holy Week Fair of Santiago de Huari in Bolivia. During these years, I also visited several markets and stalls in Tilcara (Jujuy, Argentina), Lima, Cusco, Huánuco, Trujillo, Chiclayo, Iquitos (Peru), San Pedro de Atacama (Chile), and Sucre (Bolivia), without finding *vilca* seeds alone or in preparations.

In each market or fair, all stallholders selling Anadenanthera, which are not usually many, were interviewed. In all cases, only one stall was found, except in the Central Market of Potosí, where there were two, and in the Santiago de Wari fair, where there were five. The number was obtained by direct observation of plant remains at the stalls and also by asking the local population who sell the plant (using the particular name given to the plant in each place and also showing its seed). However, it should be noted that both fairs and markets are dynamic and change from year to year, and the number of vendors may vary as well as the presence or absence of products, including *vilca*. No formal interviews were conducted, instead a friendly conversation was sought, explaining my research interests. In the course of the conversation, the name of the plant, the parts used, its place of origin, and its applications were discussed. The records from these visits were systematized and compared with the results obtained from the literature.

The results of the bibliographic research are presented below, followed by the findings of the fieldwork.

## 3. Results

### 3.1. Bibliographic Results

**Figure 2:**
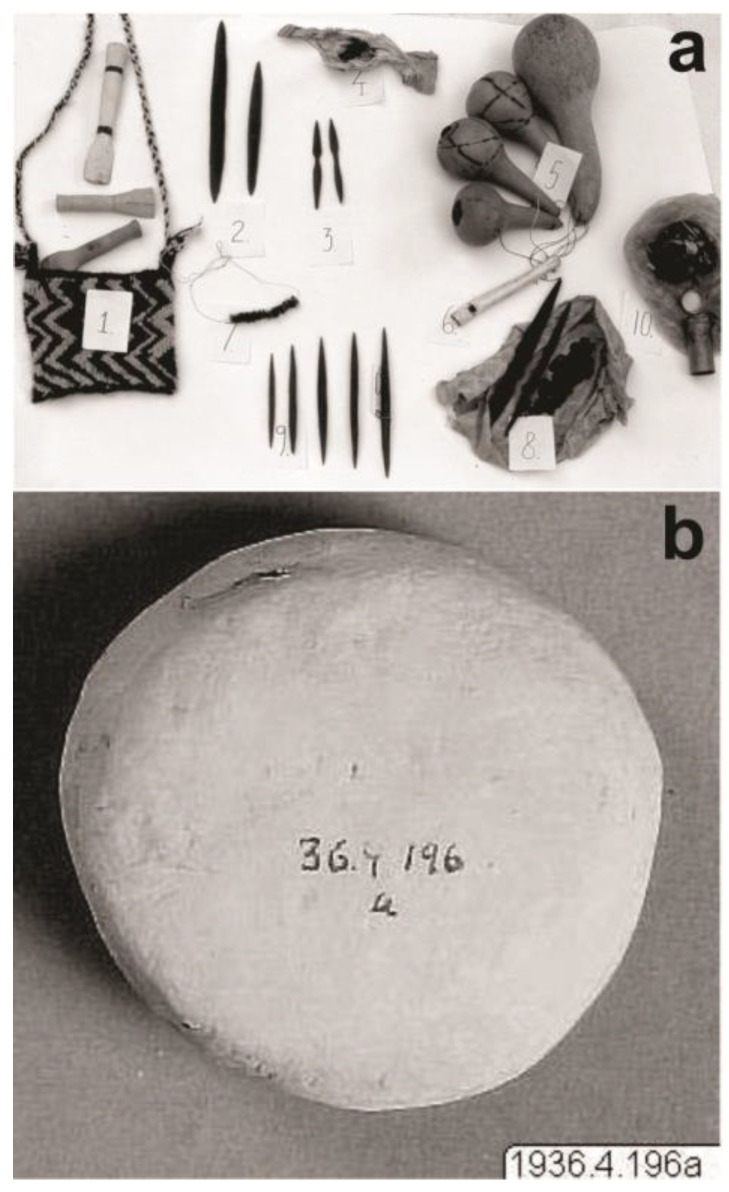
‘Weenhayek shaman’s implements (**a**) 1—bag with ceramic and wooden pipes; 2—large vehicles for spirits; 3—small vehicles for spirits; 4—“secret”; 5—gourds; 6—heron’s whistle; 7—cebil seed bracelet; 8—cebil seeds and large vehicles; 9—set of vehicles; 10—coca and a jar of bicarbonate ([33], Figure 6). (**b**) ‘Weenhayek plate for inhaling cebil ([32], Figure 38).

The inhalation of snuff made from *vilca* seeds was recently documented by Gili [8] as a healing practice performed by a descendant of the Mochica people of the northern coast of Peru. Among Wichí shamans, the inhalation and smoking of *cebil* seeds are established practices that enable them to obtain visions, perform healings, and communicate with spirits [29,30,31,32,33,34,35]. Field records from the 1970 decade among the ‘Weenhayek (Mataco-Mak’á linguistic family) settled in the Boreal Chaco, on both sides of the border between Bolivia (Tarija) and Argentina (Salta) provide interesting information. The seeds are harvested once a year, dried (sometimes roasted), strung on a fine thread made from *Bromelia* [Bromeliaceae], and stored as “bracelets” (34 cm long). These “bracelets” are of considerable value and are traded to regions where the tree is rare (Figure 2a). When *hataaj* (wichí name for *cebil*) powder is needed, the dried seeds are roasted, ground and the powder is put directly into the nostrils. There are records from the 1930s of a clay container called *hataaj’wet* (meaning ‘the cebil, its place’) being used as a base for grinding and/or snuff inhalation (Figure 2b). Also recorded in that decade is the use of a gourd for grinding, from which shamans would then take “pinches” to inhale during ceremonies. The ground cebil is also mixed with tobacco to be smoked in pipes (Figure 2a) [32,33].

The Chorote shamans (Mataco–Mataguayo linguistic family) of eastern Salta also use the *cebil*, a practice they acquired from the Wichí. Particularly, female shamans (*aiewu-ki*), who have the chant of this plant, make women burn its fruits to ashes when they wish to kill another woman [36].

In the south of Peru and Bolivia, north of Chile and northwest Argentina, other uses exist, which will be detailed below. Notably, in northern Chile and Argentina, historical records and testimonies from decades ago refer to the *yungas* as suppliers of *cebil* or *vilca* [8,37,38]. These members of the Kallawaya ethnic group, recognized as itinerant healers, are also known as *yungueños* [39].

In addition to the uses detailed below and summarized in Table 1, *cebil* bark has been widely used in leather tanning from colonial times to the present in several parts of northwest Argentina, such as eastern Catamarca. Here, veterinary uses of the bark and forage uses of leaves and fruits (for animals introduced post-conquest) are reported along with artisanal and fuel uses of the wood [40,41,42,43]. Among the Wichí, the bark, rich in tannins, is also used to tan hides and dye fibers [3], and the powder of ground seeds is placed on the eyelids to cure conjunctivitis; care must be taken to ensure that the powder does not enter the eyes as it may damage them [35]. Additionally, there is a use of *cebil* seeds reported as “magical”, where the victim of a dog bite can kill the aggressor animal by putting ground seeds in its food [44]. According to Suárez, “some seeds are ground and the powder is placed on the wound. This helps the wound to heal and at the same time the ‘dog’ that caused the wound becomes sick, completely inappetent and eventually dies” [35].

In the vicinity of Cuzco, Lira mentions the administration of ground *willka willka* (*A. colubrina*) seeds in the food of dogs to make them fierce [45]. Cooper notes that the Piro and Catawishi (from the southern Amazonia in Peru) administer Anadenanthera, apparently as snuff, to hunting dogs to make them more alert and improve their vision [6]. Infusions made from *cebil* leaves are known to induce heat in cows [46], while consumption of the immature fruits can be abortive in large animals [43].

In eastern Salta (northwest Argentina), an infusion of *cebil* bark with other ingredients is used as an external bath to cure skin diseases and other “hot” conditions [47]. The Wichí also make a decoction of the bark for stomach pains and make enemas with unripe pods, sometimes adding leaves to treat headaches [1,3]. De Lucca and Zalles report that in Bolivia, a decoction of the leaves is consumed as an excellent tonic for the stomach and womb. A decoction of the bark, taken as a drink, is effective against diarrhea and is also used in lavages to stop bleeding. Additionally, both the bark and resin are utilized for treating lung ailments and bronchitis [46].

In the following sections, we will examine the documented uses of *Anadenanthera colubrina* var. *cebil* seeds in the context of human consumption.

**Table 1:** Preparation and administration methods of *Anadenanthera colubrina* reported for human consumption in the southern Andean area.

Plant Part	Preparation and Consumption	Combination with Other Substances	Chronology	Geographical Area		Bibliography
Seed	Burnt ground (smoked)	*Nicotiana*, *Nicotiana tabacum* L. [Solanaceae]	Pre-Hispanic (ca 2000–0 BC)	Northwest Argentina		[4,13,26]
		*Nicotiana tabacum*, “aromo” (*Acacia* and *Vachellia* genus, Fabaceae)	Modern	Wichi, Salta and Formosa, Argentina; ‘Weenhayek in Tarija, Bolivia and Salta, Argentina		[1,31,32,33,34,35]
	Ground (inhaled)	*Nicotiana*	Pre-Hispanic (ca BC 1000–BC 1000)	Peru, Bolivia, northern Chile, and northwest Argentina		[3,10,12,15]
			Colonial 16th and 17th centuries	Lules from Santiago del Estero and Indians from Córdoba, Argentina		[24]
			Modern	Mochica in the northern Peruvian coast; Wichi in Salta and Formosa, Argentina; ‘Weenhayek in Tarija, Bolivia and Salta, Argentina		[8,29,33,34,35]
	Ground (placed on the organ concerned)		Modern	Wichi, Salta, Argentina		[35]
	Ground in liquid suspension (enema)		Pre-Hispanic (ca. AD 500–1500)	Bolivia; northern Chile; Jujuy, Argentina		[14]
			Colonial 17th century	Reference to Inca customs		Guaman Poma 1615 in [14]
	Whole in fermented drink (beverage)	In “chicha de molle” (*Schinus molle*)	Pre-Hispanic (ca. BC 600–1000)	Southern Peru (Wari sites)		[16]
	Not specified whether whole or ground in fermented drink (beverage)	In “aloja” (fermented *Neltuma* pods)	Pre-Hispanic (1000–770 BC)		Northern Chile	[17]
		In chicha	Colonial 16th century		Peru	Bernabé Cobo 1563 and Polo de Ondegardo 1571 in [23]
	Not specified whether whole or ground in infusion (beverage)	In “Polipodio” (*Polypodium* fern) root decoction				Bernabé Cobo 1563 in [23]
	Ground in hot water (beverage)		Colonial 18th century	Santiago del Estero, Argentina		[28]
	Ground as infusion (beverage)	In “charcoal tea” (remains of smoked elements), with basil (*Ocimum basilicum* L.-Lamiaceae) or lemon balm (*Melissa officinalis* L.-Lamiaceae)	Modern	Salta, Argentina		[37]
	Ground in distilled spirit (beverage)	Corn kernels, wayluru (*Citharexylum herrerae* Mansf.-Verbenaceae) seeds, coca seeds, a couple of carnation flowers and two mineral fragments (all ground together into a mixture called *llampu*).	Modern	Ayacucho, Peru		[48]
	Not specified whether whole or ground in water (beverage)		Colonial 16th, 17th, and 18th centuries	Juli, Bolivia		Bertonio 1612 in [49], Guaman Poma 1615 in [14]
		With honey		Peru		Bernabé Cobo 1563 in [23]
				Santiago del Estero, Argentina		[28]
			Modern	Bolivia and Bolivia–Argentina border		[38,39,50,51,52]
	Whole (buried or burned)	On ceremonial “mesas” with multiple ingredients depending on the case	Modern	Ayacucho, Peru and Bolivia		[39,53,54,55]
	Whole (worn as a charm)	Alone or wrapped in wool		Cuzco, Peru; Bolivia; northwest Argentina		[8,28,39]
	Whole (chewed)		Colonial 17th century	Pampas and Huarpes, central western Argentina		Ovalle 1646 in [5,24]
Leaf	Decoction (drink or enema)		Modern	Bolivia		[46,51]
Bark	Decoction (beverage)			Bolivia. Wichi, Argentina		[3,46]
	Decoction (baths)	Together with bark and flowers of *Vachellia caven* (Molina) Seigler and Ebinger and plants of “mastuerzo” (*Lepidium didymum*L.-Brassicaceae)	Modern	Northwest Argentina		[47]
				Bolivia		[46]
Resin	Probably beverage		Modern	Bolivia		[46]
Pods	Burned (inhaled)		Colonial 18th century	Abipones, Formosa, Argentina		Dobrizhoffer 1784 in [5]
	Decoction of unripe pods (baths)	*A. colubrina* var *cebil* leaves	Modern	Wichi, Argentina		[1,3]

#### 3.1.1. For Protection (*Contra*) and to Attract Good Luck

In the first half of the 20th century, testimonies from Santiago del Estero and Catamarca, Argentina, describe the use of *cebil* seeds as amulets and charms for protection (called *contra*) to ward off witch attacks [28,43]. Local testimonies indicate that wearing three seeds in the hem of trousers or a dress, or a necklace hidden in the clothing, was believed to prevent the influence of witchcraft [43]. In northern Chile, *vilca* seeds from Bolivia wrapped in *pante* wool (dyed fleece of ritual value) are also used as amulets [8]. In Bolivia, *Yungeño* traders sell *vilca* seeds for good luck and remedies for discomfort caused by envy [39]. According to records from the 1980s, Pellegrin noted the sale of *vilca* seeds on the border between La Quiaca (Argentina) and Villazón (Bolivia) [38]. These seeds are either carried (*se trajinan*) as amulets or made into an infusion to be drunk three times a day for good luck.

From Salta to Villazón, *cebil* seeds are used as amulets to counteract evils and attract luck or fortune. These elements, known as *cuti* in Andean culture, embody the idea of turning or changing direction. This concept involves redirecting negative forces rather than undoing them. Many *cuti* elements, including certain fruits, have a counterclockwise twist that stands for this idea [55,56,57]. *Cuti* includes various plant elements, such as *wuayruros* (usually *Ormosia coccinea* (Aubl.) Jacks. [Fabaceae] seeds) and *willcacuti* (*vilca* seeds), which must be used in specific numbers or proportions to be effective. They can function independently or be combined with other elements by ritual specialists. In the latter case, they can be bought in markets already prepared in small bags, glass containers, or *chuspas* (small traditional woven bags), which must be carried by the individual [57].

Gentile analyzed stalls selling traditional Andean talismans and amulets across various provinces in Argentina and in Sucre (Bolivia) [58]. According to Gentile, talismans are crafted to attract or obtain desired outcomes. They are made up of various elements and require preparation by a ritualist who “activates” them, endowing the talismans with their own will, requiring regular attention. Amulets, on the other hand, are unmodified animal, plant, or mineral parts that can be purchased or found.

Gentile highlights the predominance of plant elements in the creation of talismans, mainly *wuayruros*, followed by *mastuerzo* or *cuti* (*Strombocarpa strombulifera* (Lam.) A. Gray., Fabaceae) and *cebil* seeds. She describes a composite talisman acquired in Sucre in the 1990s intended to attract a loved one, which included multiple ingredients, including *vilca* seeds. Metraux also notes that in Bolivia, seeds of the *wilka* or *wilkacipa* legume, or the fruit of the *Prosopis strobolifera* (*Strombocarpa strombulifera*), known as *kutiwainito*, are drunk to counter curses and ailments like neck pain [50].

#### 3.1.2. As Purge, Cleanser, and Medicine

The main chroniclers of the central Andes in the 17th century often mentioned the use of *vilca* as a purge, administered either as an enema or as a drink with *chicha*, due to its laxative and emetic properties (Table 1). *Vilca* was also used to cleanse the chest and stomach and to stimulate urination by drinking its seeds cooked with honey. Additionally, it was believed to enhance fertility in women [23]. Larraín explains that “to purge” meant to expel “bad humours”, a practice achieved in the past, as it appears in early chronicles, through the inhalation and oral consumption of *vilca* for prophylactic therapeutic purposes [59]. This cleansing process facilitated the plant’s stimulating and ritualistic effects, allowing the sacred to manifest within the individual, as *willka* also means “sacred”. In this context, the effects of *vilca* are similar to those defined for entheogens [2].

The use of *vilca vilca* as a stimulant and aphrodisiac is documented in the 19th century *Callahuayan* pharmacopeia [60]. The cooked leaf, either in a decoction or enema, is used to cleanse the chest, stomach, and womb of parasites and mucus and to provoke menstruation. In [3], Yacovleff and Herrera 1934–1935 note that *cebil* seeds are sold as a laxative in Peruvian markets. In northern Chile, the seeds are currently used “as a remedy by women in the Salado river basin” [49]. In Bolivia, *vilca vilca* is used as an “aphrodisiac stimulant; it is taken as *mate* (infusion) to treat sterility and impotence, and to alleviate anger and melancholy” [51]. Karen Urcia of the Mochica people explains that the inhaled seed powder “is used for cleansing, as it is considered the strongest plant capable of expelling deep-seated evils” [8]. In San Pedro de Atacama, Chile, seeds wrapped in wool or red fleece are used to cleanse individuals suffering from illnesses [8].

According to Loza and Quispe, traditional Aymara midwives in Bolivia use *vilca* seeds, administered in liquid form, to facilitate loosening of the placenta [39]. These seeds are sold in markets in the city of La Paz (specifically on Santa Cruz and Linares streets) and in El Alto (in La Ceja area). They are marketed as an abortifacient and for inducing menstruation when prepared as a decoction [52].

#### 3.1.3. To Cure *Susto* (*Susto* (Fright) Refers to a Disease Caused by the Loss of the Vital Principle, or *animu*, of the Person, Which Leaves the Body and Becomes Retained Somewhere) and Restoration of the Person’s Vital Principle (The Cure for *Susto* Involves the Return of the Retained or “grabbed” *animu* to the Individual’s Body)

Ethnographic records from the 1970s and 1990s in Molinos, Salta (northwest Argentina), document the use of *vilca* to cure *susto* in children [61]. The sick children are smoked (*se sahuman*) with palm, blessed cane, incense, and lamb’s wool. Subsequently, the combustion residue is used to prepare an infusion, to which ground *vilca* is added. This preparation is typically conducted collaboratively by the “medicine woman” and the affected person’s family.

Local testimonies inform additional methods for treating *susto*. One approach involves taking a fourth of a *vilca* seed, burning it alongside with a hummingbird’s nest and a piece of condor feather. Furthermore, three drops of holy water and earth from the afflicted child’s home are included. Alternatively, basil, lemon balm, and *vilca* ground with a spoon and a small, clean stone, are blended, strained, and administered to the frightened individual [37]. Moreover, in Molinos, *vilca* is combined with various ingredients to concoct infusions aimed at alleviating heart maladies attributed to bad wishes [62].

In the case of the Department of Ayacucho, in Peru, *willka* seeds are integral to various ritual bundles called *pagapu* (payments). These bundles, comprising several ingredients, are buried in the place where the individual was “grabbed” by the earth or *Pacha*, often in a cemetery or a burial ground of *gentiles* (*Gentiles* refers to a pre-human generation associated with the underworld). This ritual is performed to compel the place to release the *animu* taken from the person, thereby curing them once this vital essence returns to their body [53]. Additionally, in Ayacucho, a handful of *willka* seeds is commonly used as an ingredient in *mesas* or *pagapus* to treat ailments believed to be caused by *Pacha.* These earth-related diseases may have various origins [63].

#### 3.1.4. As an Ingredient in *Mesas* (Ritual Bundles)

As we have seen, *vilca* and *wuayruros* are *cutis* and often appear together, commonly featured in the so-called *black mesas*. According to Fernández Juárez, this type of *mesa* is used to cleanse, repel, and return the patient’s affliction through a “decontaminating” preparation that involves “inversion movements” [54]. Rösing adds that these *mesas* with *kutis* operate a “change of direction” through both the ingredients used and the counterclockwise gestures performed by the specialist [55]. This practice aims to dispel sorrows and sadness while ensuring that sacred places receive the offering and reciprocate, a notion closely linked to the concept of Andean reciprocity. Consequently, protection and rejection are seen as exercises in the constant return of what arrives and must be sent away to make room for what is to be attracted.

*Vilca* seeds are used in various ritual *mesas*, along with many other ingredients, among the Aymara groups of the Bolivian Altiplano. During his travels in 1930 and 1931, Metraux documented their use in *saxra mesas*, which are employed against curses and to treat diseases caused by “the air” or “blows”, as well as in *chywchy mesa* composed of *chywchys* (lead miniatures) and *wuayruru* seeds [64].

Fernández Juárez explains that *chiwchis* are lead and tin miniatures of domestic objects, people, animals, tools, crosses, and celestial bodies (such as stars and the moon), crafted from shiny papers, along with *willka* and *wuayruru* seeds [65]. These elements form the *chiwchi mesa* but they are also components of other *mesas,* like the *gloria mesa*, which is associated with celestial entities, such as virgins and saints or with lightning [65]. Nordenskiöld describes the objects buried when a house is built and mentions that, in addition to miniatures, *vilca* and *huayruros* seeds are acquired in the La Paz market for this purpose [66].

Among the Aymara ritual specialists of La Paz and El Alto, Bolivia, Loza and Quispe mention *vilca* seeds as part of the *castilla mesa*, which is associated with the conservation of life and protection against threats from evil entities inhabiting the cosmos [39]. In the Central Market of San Pedro de Cusco, Peru, *vilca* is sold for use in dispatch (*despacho)*, an offering incinerated to honor specific entities [8]. In Humamarca, Bolivia, *willka* seeds are included in the *saminchay* or “smoke offerings”, along with incense, gold and pepper, coca seeds, and llama fat. These offerings are presented to sacred sites to release water for irrigating fields [67].

#### 3.1.5. As a Ritual Drink

As previously discussed, the inclusion of *vilca* in *chicha* is mentioned by early chroniclers and has archeological antecedents in northern Chile and the Cuzco area. Torres explored this topic by examining the chemistry behind this combination as well as its historical trajectory and continued practice today [23]. Torres highlighted a unique case: “*cebil* wine” among the Wichí, referencing the ethnographic work of Califano [30]. Califano recounts a shamanic initiation where the individual visits the world of spirits, a place where the *jataj* or *cebil* seed is a wine that produces similar effects once the initiate drinks it. This narrative appears to be a case of perspectivism [68], which is frequent in several South American psychoactive plants [69,70], although it had not been previously noted in the case of *Anadenanthera*.

Another contemporary account is provided by Isbell. In her ethnography conducted in Chuschi (Department of Ayacucho, Peru) in August [48], she details the preparation of a ritual drink called *llampu*, which includes *willca* seeds and is used for rituals associated with *herranza* (livestock branding). This drink, in addition to ground *vilca* seeds, incorporates pairs of pulverized ingredients: large-grain white corn kernels, *wayluru* seeds *(Citharexylum herrerae* Mansf., Verbenaceae), coca seeds, white carnation flowers, and fragments of minerals known as red and white *llampu*, along with “crude gold” and “crude silver”. The term *llampu* seems to refer both to the reddish-colored drink and to certain minerals included in its preparation, as well as to the ground powder of the mentioned ingredients. This ground preparation, along with some of its ingredients, such as *vilca* seeds, are kept inside the ritual bundles and other paraphernalia for the *herranza* ceremony.

The drink is offered as payment (poured on the ground) to the *wamanis* (powerful mountains) and is consumed as a purifying agent for ritual preparation and as protection against contamination, capture, and disease caused by the *wamanis*. As Isbell states:

“When the *llampu* preparation was finished, our compadre poured *trago* into a shell and sprinkled *llampu* over the liquid, then a small quantity of *achita* (*Chenopodium pallidicaule*). He prepared an identical mixture in a horn cup and drank from both. We followed him by drinking the double concoctions in turn. He explained to us that the double shots would protect us from the *Wamani*” [48].

**Figure 3:**
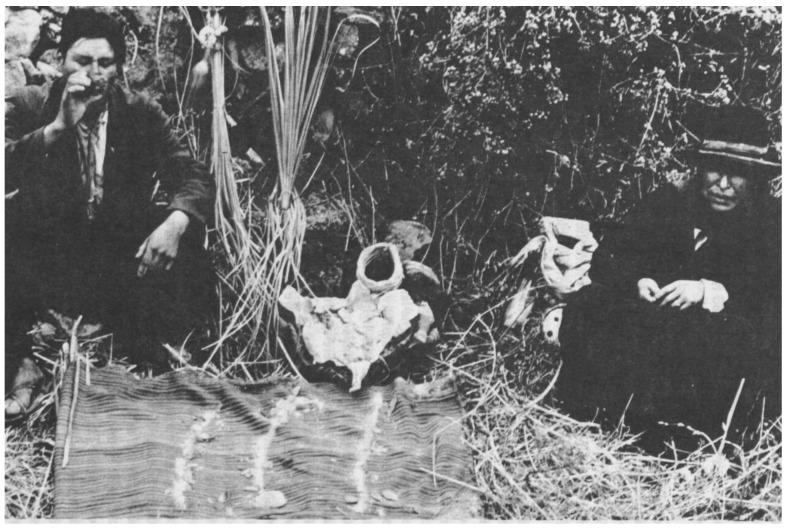
Ritual bundle (*mesa*) during *herranza* rituals in Chuschi, Peru. Lines made of *llampu*, a ceremonial powder that includes *vilca* ([48], plate 12).

These two beverages are consumed by everyone who participates in the *herranza* ritual. In addition to the preparation of the drink, the ground *llampu* is essential when setting up the ceremonial *mesa* next to the corral:

“The larger cloth of red *llampu* was untied and the figurines, the *illas*, were carefully set upright in the *llampu.* Another cloth contained a sea shell filled with lighter-colored *llampu*, several fossil shells, and some red wool. Our compadre sprinkled three lines of *llampu* on the Poncho, then coca leaves were carefully scattered along the lines. The small knife was dipped in *llampu* and placed near the center line. The chunks of “crude gold” and “crude silver” were stationed on the other lines. Now the ceremonial *mesa* was complete” [48] (Figure 3).

After arranging the *mesa*, the animals are brought into the corral. The rite officiant then sprays *llampu* into the air, tracing a counterclockwise path. Once the branding is complete and the animals leave the corral, they are also sprayed with *llampu* [48].

### 3.2. Fieldwork Results (2017–2019)

#### 3.2.1. Names

Although the names given to *Anadenanthera colubrina* var. *cebil* seeds were briefly mentioned at the beginning of this paper, we will delve deeper into them in this section. During our fieldwork, we recorded several names that were not included in the existing bibliography (Table 2).

*Wilka*, *Villca*, or *Vilca* is an Aymara word which, according to several chroniclers, refers to the ancient name of the sun before it was called *Inti*. It is also the name of certain idols and shrines and refers to sacred beings sometimes synonymous with *huaca.* Additionally, it is the name of a tree whose fruits are used as medicine or visionary vehicle, as previously mentioned [3,7].

Cárdenas, in his manual of economic plants in Bolivia [71], mentions other names for that species: *curupau* from the east, *yarisana* from the Yungas, *villca* from Peru, and *cebil* from Argentina. Altschul, in addition to *vilca* and *cebil* with their variants and *curupau*, also mentions *curupai* for Paraguay [5]. Oblitas Poblete also cites the name *bayan* [51].

In [39], Giraul 1988 says that the Aymara use the term *willka, chipa,* or *kipi*. The latter name resonates with *chipi*, recorded by Forgione in 1983 in the Villazón market, referring to Anadenanthera seeds from the Bolivian yungas [38]. During my fieldwork, I most frequently recorded the term *chipi*, albeit with different intonations: *chi’j’pi* in the Central Market of Potosí, *chipi* in the Vicuñas Market of Potosí and in Tarabuco, and *ch’ij’pi* in the Santiago de Huari Fair.

In addition to the names mentioned above, there are others not found in the literature: *liman* in the Carhuaz Market (Peru) and *urilimpi* in the San Salvador de Jujuy Market (Argentina). In the Mercado Modelo of Chiclayo (Peru), whose sector of ritual and medicinal plants is known in tourist jargon as *Mercado de los Brujos* or *de las Huaringas* (the latter name alluding to some famous high Andean lagoons of great power), one of the vendors told me when I asked him about the *urilimpi* that they did not have it. They bring it from the jungle and sometimes they have it, but very rarely. Therefore, although I could not confirm its botanical identity, the vernacular name was recognized locally.

#### 3.2.2. Source and Vendors

Regarding the sourcing of the seeds sold in markets and fairs, those in Potosí come from Cochabamba, Santa Cruz, or Argentina. In Tarabuco, they are brought from “the valleys”, referring to the area of Tarija and Oruro. In San Salvador de Jujuy, the seeds come from Peru, enter Bolivia through Desaguadero, and then reach the province of Jujuy. These references to places of origin should not always be taken literally. Access to these seeds is closely linked to the figure of *yungas* or *Kallawaya* doctors. Referring to them or their area of residence as the source of the seeds adds value. Even in Molinos, Salta, people prefer seeds sold by the *yungas*, as they are considered more potent and effective than those they could collect themselves (Pochettino, personal report).

#### 3.2.3. Uses

**Figure 4:**
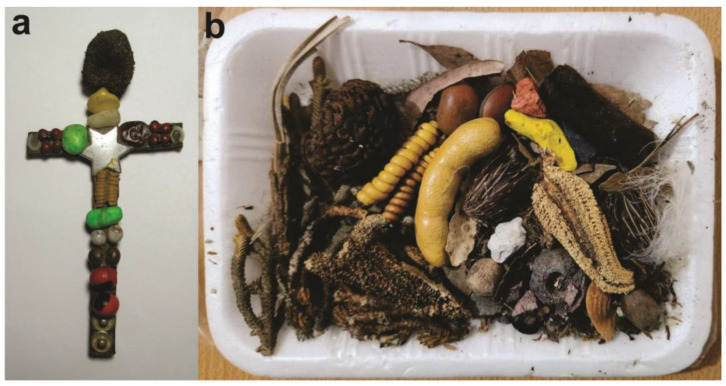
(**a**) Cross purchased in 2018 at the market of Carhuaz, Peru. (**b**) Ritual bundle for cleansing baths, bought in the old market of San Salvador de Jujuy, 2016. Photos taken by the author.

In the market of Carhuaz, Peru, I acquired a cross (Figure 4a) used both for protection of the house and livestock and to attract money and fortune. This cross is possibly made of *chonta* palm (*Iriartea deltoidea* Ruiz & Pav. or *Bactris gasipaes* Kunth., both of the Arecaceae family), which is used in the Andes to craft other powerful objects, such as ceremonial staffs. It is associated with jungle groups like the *Chunchos* in religious dances from Paucartambo, Cusco, to northern Chile [72]. This cross features *cebil* and *wuayruro* seeds, *mastuerzo* fruits—all of them *kuti* plants—along with a small horseshoe and metal studs. When I acquired it, the seller had me pass it through iron filings so that the stone magnet at its upper part would materialize the idea of attracting with my hand [58]. In the old market of San Salvador de Jujuy, I was told that the seeds are “for luck” due to their coin-like shape. They are part of a ritual bundle for cleansing baths, which also come from Peru via Bolivia and are sold in smaller quantities in Jujuy (Figure 4b).

**Figure 5:**
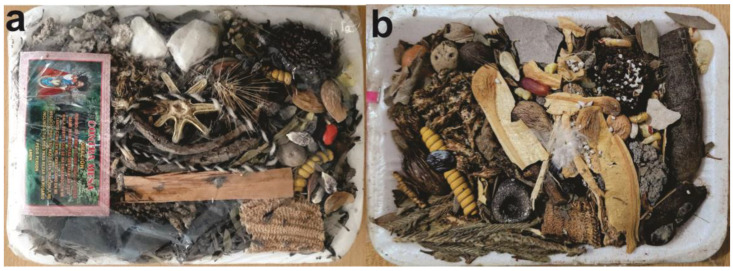
(**a**) *Contra mesa* purchased in the central market of Potosí, Bolivia, 2017; (**b**) similar bundle prepared in the market of Cochabamba, Bolivia, 2017. Photos by the author.

In the central market of Potosí, *vilca* seeds are sold individually or as part of the *contra mesa* (Figure 5a), which is used to wash and cleanse oneself of all spiritual harm and dirt. The residue must be discarded in an open place without looking back. This kit even includes an image of Saint James (*San Santiago*), a saint linked to the pre-Hispanic deity *Illapa*, associated with lightning and thunder, along with a brief prayer. In the Cochabamba market, a similar preparation with *vilca* seeds is sold for the same purpose; although, it is not referred to as *black mesa* (Figure 5b).

**Figure 6:**
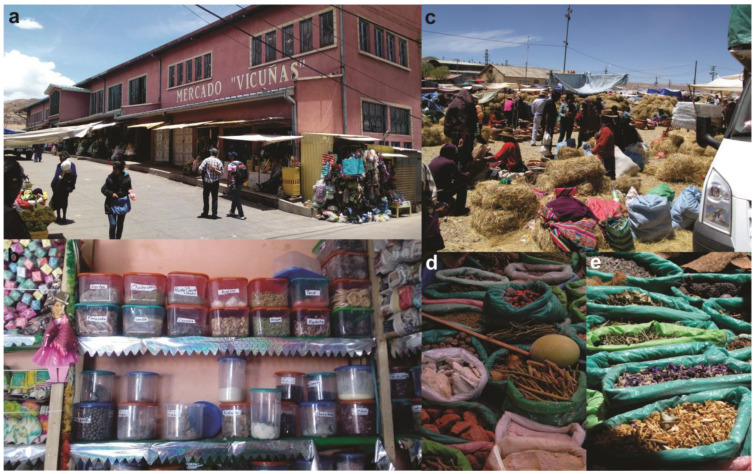
(**a**,**b**) Vicuñas Market, Bolivia, 2017. (**c**–**e**) Manka Fiesta, Argentina, 2013. Photos by the author.

In Bolivia, at the Vicuñas Market (Figure 6a,b), *willka* is available either as loose seeds or as a powdered preparation meant to be dissolved in a liter of boiled water and consumed three times a day. This remedy is used to treat *susto* and womb disorders. Similarly, at the Holy Week Fair in Santiago de Huari, vendors sell *willka* seeds for the same purposes. According to one vendor, “you put a couple of seeds in water and the woman takes it”.

To summarize, in markets across Peru (Carhuaz), Bolivia (Cochabamba), and northwest Argentina (Manka fiesta) (Figure 6c–e), *vilca* seeds are sold as protection and *contra* or *kuti*. They are available alone, combined with other elements, or placed on supports like crosses. *Vilca* seeds are also sold to attract luck (*suerte*), cleanse from damage (*daño*) and spiritual evils, cure *susto*, and heal the womb (Table 2).

The relationship between all the uses of *vilca* seeds reported in this paper may initially appear confusing or unrelated. In the next section, I will delve into an argumentation that aims to elucidate the connections between the various reported uses of *vilca* seeds. Drawing from native explanations and Andean perspectives on the body, illness, and healing, I will elucidate how these seemingly unrelated uses are interconnected. This discussion will provide clarity on the cultural and traditional rationale behind the multifaceted therapeutic applications of *vilca* seeds across different Andean regions.

## 4. Discussion

The information gathered from the literature and ethnobotanical studies in south Andean markets revealed both commonalities and variations in the uses, names, and descriptions of *Anadenanthera* seeds. The diversity of applications and their seemingly unconnected nature can be attributed to Andean concepts of illness and the underlying dynamics at play. By considering these cultural perspectives, we can interpret the contemporary therapeutic uses of *vilca* or *cebil* in terms of “movements” as integral to the healing process.

Firstly, the use of *Anadenanthera* seeds reflects a fundamental concept of movement—specifically, the actions of releasing and attracting. These seeds are associated with purgative and abortive properties, indicating their ability to expel substances from the body, such as phlegm, mucus, the placenta, or to induce menstruation. This concept extends beyond physical purging; it also encompasses spiritual cleansing rituals, known as *limpias*, which aim to release evil energies held within a patient. Moreover, the idea of releasing is integral to dispatches made to entities in the landscape. These offerings seek to persuade those entities to release trapped waters for irrigating crop fields.

The treatment of *susto*, a condition where the patient’s soul is trapped by a non-human entity, further exemplifies the need for releasing and attracting movements in healing. The goal is to release the trapped soul so that it may return to the patient’s body. Attracting is crucial for healing processes, the aim is to attract desired elements—whether it be the patient’s soul for healing or qualities like fortune, health, or love. This involves not only removing obstacles that hinder these qualities but also actively working to maintain their presence and prevent them from turning towards misfortune due to external human or non-human influences.

Secondly, we found the movements of stopping and returning, which underscores the use of *vilca* as *kuti* or *contra* to ward off harm. This practice involves redirecting damage back to its origin rather than attempting to nullify or eradicate it completely, as diseases and misfortunes emanate from a specific source, human or otherwise [55]. This approach acknowledges the kinetic nature of afflictions, where new sources of harm can continue to emerge or return. This also explains why *vilca* seeds act as protective amulets, playing a dual role: shielding the body to prevent the displacement of its spirit and embodying a continuous, rotation movement. This rotational motion compels incoming negativity to reverse its trajectory and to return to its point of origin. Many plant elements achieve this effect through counterclockwise twisting [56]. Although the shape of the *vilca* seed itself does not explicitly suggest twisting or turning, its shape significantly influences its therapeutic efficacy.

Lira describes *Anadenanthera* seeds, known as *Willka Willka*, pointing out the following:

“The center of the almond has a human footprint shape in some cases, and in others, the form of a horseshoe, a cow’s foot, or a sheep’s foot. In other cases, it has the shape of a field plot (*chacra*), a dog, or a road. Thus, the use of the almond depends on its shape. If it has the shape of a human foot, it is buried in the ground to prevent servants from leaving a household. The dog-shaped almond is buried at the entrance of a farm or farmhouse to prevent any mastiffs from dying (…). The almond with a horseshoe shape is buried in the manger or in the horses’ stall to protect them from death or theft. The same applies to almonds shaped like a cow’s foot, a sheep’s foot, or a *chacra*, which are arranged in the same way” [45].

Lira then describes how the seeds are buried along with coca leaves, fat, aromatic plants, and other vegetables. His work emphasizes the importance of the seed’s shape and its fissural line, which helps to retain those entities (humans, animals, field plots) with morphological similarity and fend off (or return) potential harm. The implications of the seed’s shape for its healing properties are uniquely detailed in Lira’s work and were corroborated during field research.

For instance, in the former market of San Salvador de Jujuy, I was told that the *cebil* seed attracts luck due to its resemblance to a coin. This idea aligns with observations made by early chroniclers such as Bernabé Cobo, who likened it to a half-real, and Cristóbal de Albornoz, who compared it to a copper coin of Castile [23]. This resemblance to coins that entered America during colonization adds new significance to the *vilca* seed, which became part of the transactions between Spaniards or Creoles and indigenous chiefs in Argentina. Likewise, ancient coins are still used today in Kallawaya ritual bundles and are sometimes considered as *illas* [73], underscoring the enduring cultural and symbolic values of these seeds.

Lira mentions ritual bundles intended to change bad luck, which include ingredients such as *wilka* and *wuayruro* seeds, as well as “a small steel cross, three old silver half coins, seven *reales*, seven *pesetas*, and seven current half coins” [45]. Additionally, the fissural line inside the seed, which resembles a horseshoe, is linked not only to the hoof of many livestock animals but also to the horseshoe itself. This is another symbol known to attract or retain fortune. As noted, *vilca* seed is accompanied by a miniature metal horseshoe in the cross of Carhuaz, reinforcing its role as a powerful amulet.

## 5. Conclusions

Nine uses of *Anadenanthera colubrina* var *cebil* have been recorded in the southern central Andes from the pre-Hispanic past to the present: magical–religious, medicinal, veterinary, construction, fuel, fodder, dyeing, tanning, and artifact making. These uses span almost all parts of the tree, including seeds, fruits (pods), bark, wood, resin, and leaves. The records, which may be incomplete due to gaps in documentation across the extensive geographic region, reflect a broad and integrated utilization of the tree. The therapeutic use of the plant parts, apart from the seeds, is mostly modern (from the 19th century onwards). This intensification of the uses of *Anadenanthera* trees could be due to difficulties in accessing other traditional medicines under national legislation or it could also be the result of more detailed ethnobotanical and anthropological research in modern times.

In addition to this diverse and integral use, there is a significant variation in the preparation and administration of *vilca* or *cebil* seeds for therapeutic and psychoactive or visionary purposes, with this variety in seed preparation and administration expanding after the Spanish conquest. The records reported and analyzed in this work demonstrate the ongoing use of *vilca* or *cebil* and its centrality in contemporary south Andean therapeutics, highlighting the enduring ritual value of this plant.

## Figures and Tables

**Table 2:** Results from the fieldwork regarding *Anadenanthera colubrina* var cebil *seeds*.

Country	Place	Name	Uses	Preparation Mode of Seeds	Consumption Mode
**Perú**	Ancash (Carhuaz Market)	*liman*	protection of the house and livestock; to attract money and fortune	as part of a cross	hanging in the house or business
**Bolivia**	Potosi (Central Market)	*chi’j’pi*	cleanse oneself of all spiritual harm and dirt	ritual bundle	cleansing baths
	Potosi (Vicuñas Market)	*chipi*	to treat *susto* and womb disorders	powdered or loose seeds dissolved in one liter of boiled water	drunk three times a day
	Cochabamba (Cochabamba Market)	*chi’j’pi*	cleanse oneself of all spiritual harm and dirt	ritual bundle	cleansing baths
	Tarabuco (Tarabuco Market)	*chipi*	to treat *susto* and womb disorders	in warm water	drunk
	Santiago de Huari (Holy Week Fair)	*ch’ij’pi*	to treat *susto* and womb disorders	two seeds in water	drunk
**Argentina**	San Salvador de Jujuy (old bus terminal market)	*urilimpi*	for luck	ritual bundle	cleansing baths
	La Quiaca (Manka Fiesta fair)	*vilca*	as protection against spiritual harm	powdered to dissolve in water	drunk

## Data Availability

People interviewed in the markets agreed to share information with our research project and to make public the information provided in this manuscript. The vouchers specimens are housed in the Institute of Anthropology of Córdoba (IDACOR-CONICET).
